# Tissue levels of persistent organic pollutants and biotransformation enzyme polymorphisms in human stomach cancer

**DOI:** 10.1007/s10552-026-02154-z

**Published:** 2026-03-24

**Authors:** Rasih Kocagöz, İlgen Onat, Merve Demirbügen Öz, Koray Atilla, Halit Sinan Süzen, Hilmi Orhan

**Affiliations:** 1 Department of Pharmaceutical Toxicology, Faculty of Pharmacy, Ege University, Bornova, 35040 İzmir, Türkiye; 2 Department of Pharmaceutical Toxicology, Faculty of Pharmacy, Ankara University, Tandoğan, 06350 Ankara, Türkiye; 3 Department of General Surgery, Faculty of Medicine, Dokuz Eylül University, İnciraltı, 35340 İzmir, Türkiye

**Keywords:** Persistent organic pollutants, Gastric cancer, Gastric tissue, Organochlorine pesticides, Polychlorinated biphenyls, Genetic polymorphism, Oxidative stress, Biotransformation enzymes

## Abstract

**Purpose:**

Persistent organic pollutants (POPs) are lipophilic environmental contaminants with carcinogenic potential, yet data on their accumulation in human gastric tissue are limited. This study aimed to comprehensively quantify 32 POPs in malignant and non-malignant gastric tissues and to evaluate their distribution across blood and omental adipose tissue, together with metabolic enzyme activity, genetic polymorphisms, and oxidative damage markers.

**Methods:**

POP concentrations were measured in gastric tissue, blood, and omental adipose tissue from stomach cancer patients and controls undergoing stomach reduction surgery. Associations were evaluated using multivariable models adjusted for age, sex, and smoking. False discovery rate (FDR) correction was applied to account for multiple testing. Genotyping of CYP1A1, GSTP1, GSTM1, GSTT1, and OGG1 was performed, and CYP1A1 enzyme activity and urinary oxidative damage biomarkers were assessed.

**Results:**

The most robust differences were observed in gastric tissue, where 13 individual POPs and selected chemical classes remained significantly different between cancer and control groups after FDR correction. In blood, five individual POPs remained significant after correction. Several cross-compartment correlations persisted following FDR adjustment, particularly for selected HCH isomers and 4,4′-DDE. CYP1A1 activity did not differ between groups. Among oxidative biomarkers, only urinary o,o′-dityrosine remained significantly lower in cancer patients after FDR correction. In adjusted genetic models, no polymorphism demonstrated a statistically robust association in the overall analysis; the GSTP1 estimate was elevated but imprecise.

**Conclusion:**

Direct quantification of POPs in gastric tissue revealed selective accumulation patterns associated with stomach cancer. While systemic distribution and genetic variability may influence susceptibility, tissue-level POP differences represented the most consistent findings. Larger prospective studies are warranted to clarify temporal and mechanistic relationships.

**Supplementary Information:**

The online version contains supplementary material available at 10.1007/s10552-026-02154-z.

## Introduction

Stomach cancer ranks fourth among the most prevalent global cancers and third in cancer-related mortality worldwide [1, 2]. As a primary interface between ingested substances and the internal environment, the stomach is directly exposed to contaminants present in food and drinking water. Among environmental contaminants, persistent organic pollutants (POPs) are of particular concern due to their high lipophilicity, environmental persistence, bioaccumulation potential, and resistance to metabolic degradation. POPs are widely distributed in environmental compartments and food sources—including water, seafood, red meat, poultry, and vegetables—resulting in chronic low-dose human exposure. Major classes of POPs, including organochlorine pesticides (OCPs), polychlorinated biphenyls (PCBs), and polybromodiphenyl ethers (PBDEs), have been associated with several cancer types in epidemiological and experimental studies [3–6]. We recently reported significant associations between internal concentrations of POPs and kidney as well as breast cancer in human studies [7–12], and the present investigation represents the final component of this research series focusing on stomach cancer.

In 2016, the International Agency for Research on Cancer (IARC) classified polychlorinated biphenyls as Group 1 carcinogens, indicating sufficient evidence of carcinogenicity in humans. Several organochlorine pesticides and related compounds have been classified as Group 2A (probable) or Group 2B (possible) carcinogens [13]. Despite growing recognition of the carcinogenic potential of these compounds, studies examining their role in gastric carcinogenesis remain limited and inconclusive, particularly those incorporating internal dose quantification [14–16].

Most previous epidemiological investigations of POPs and stomach cancer have relied primarily on questionnaire-based exposure assessment rather than direct biological measurements [17–21]. Moreover, relatively few studies have measured POP concentrations in biological compartments closely linked to long-term storage, such as adipose tissue, and only three studies have assessed both blood and omental adipose tissue levels in gastric cancer patients [14–16]. Importantly, direct quantification of POP levels in human gastric tumor tissue has rarely been performed, limiting mechanistic interpretation and tissue-specific inference.

Given these knowledge gaps, the present study aimed to comprehensively quantify 32 POP compounds across multiple biological compartments, with primary emphasis on gastric tissue. POP concentrations were measured in both tumor and non-cancerous gastric tissues obtained from patients undergoing stomach tumor resection (cancer group) and stomach reduction surgery (control group). To our knowledge, this is the first study to directly compare a broad spectrum of POP concentrations between malignant and non-malignant gastric tissues within the same clinical framework. As secondary objectives, we evaluated POP levels in blood and omental adipose tissue to examine cross-compartment distribution patterns and assess whether circulating concentrations may partially reflect tissue burden. In addition, urinary biomarkers of oxidative damage were analyzed to explore potential systemic effects associated with POP exposure. Finally, we investigated whether genetic polymorphisms in key biotransformation and DNA repair enzymes may modify gastric cancer susceptibility in the context of environmental exposure. Cytochrome P450 1A1 (CYP1A1) and glutathione S-transferases P1, M1, and T1 (GSTP1, GSTM1, GSTT1) are phase I and phase II enzymes involved in xenobiotic metabolism, including the biotransformation of several POPs. Human 8-oxoguanine glycosylase 1 (hOGG1) plays a critical role in repairing oxidative DNA damage that may arise from reactive metabolites. The specific enzymatic activity of CYP1A1 was therefore also assessed in gastric tissue samples to provide functional context for genetic variability.

Together, this integrative approach enables evaluation of tissue-level accumulation, systemic distribution, metabolic capacity, and genetic susceptibility within a unified framework of gastric carcinogenesis.

## Material and methods

### Chemicals

Suprasolv-grade acetone, dichloromethane, n-hexane, and silica gel 60 were obtained from Merck (Germany). Organochlorine pesticide mix 3 (containing the measured OCPs), PCB mix (containing the measured PCBs), PCB-103, PCB-198, and several individual compounds were purchased from Dr. Ehrenstorfer. (YA06170100TH, USA). The PBDE mix (containing the measured PBDEs) was obtained from Wellington Laboratories. The synthesis,purification and identification of *o,o′*-dityrosine and the cold-labeled isomer *o,o′*- [ring-^2^H₆]dityrosine, used as an internal standard, were performed in-house as previously described [22]. 8-Hydroxy-2′-deoxyguanosine (8-OHdG), 3-nitro-L-tyrosine, and resorufin were obtained from Sigma-Aldrich (St. Louis, MO, USA), while 3-chloro-L-tyrosine hydrochloride was purchased from ICN Biomedicals (Zoetermeer, The Netherlands). All other reagents were of analytical grade.

### Patients and study design

Between 2015 and 2017, a total of fifty stomach cancer surgery patients and fifty stomach reduction surgery patients were recruited from the Department of General Surgery at Dokuz Eylül University. This cohort was selected for a comprehensive investigation of stomach cancer etiology. For the cancer group, eligibility was defined as a histopathologically confirmed diagnosis of stomach adenocarcinoma based on the resected tumour tissue. All eligible surgical patients during the study period were included consecutively. In the stomach reduction group, patients undergoing bariatric surgery during the same period were included consecutively, provided they had no prior diagnosis of stomach cancer. This group served as a non-cancer control group for tissue-based comparisons. The term “control” refers strictly to the absence of gastric malignancy and does not imply overall metabolic normality, as these individuals were undergoing surgery for obesity. No additional predefined inclusion or exclusion criteria (e.g., age, sex, residence, insurance status, or other clinical characteristics) were applied. Demographic data of the patients are summarized in Table S1. During surgical procedures, various biological samples were diligently collected from each patient, including blood, cancerous stomach tissue (tumour), non-cancerous stomach tissue, omental tissue, and urine. Although the samples were collected a relatively long time ago, each was analyzed within at most one year. Until analysis, the samples were stored in a –86 °C deep freezer. To enhance statistical power in detecting potential associations between enzyme genetic polymorphisms and stomach cancer development, a separate group of twenty genuinely healthy volunteers (without known chronic disease, including obesity) was included. Blood samples from these individuals were used exclusively for the determination of genotype frequencies in the investigated biotransformation and DNA repair enzyme genes; no persistent organic pollutant (POP) measurements or other biochemical analyses were performed in this group. These individuals were not included in any tissue-based or POP concentration analyses. All patients and volunteers provided written informed consent before participation. The study protocol was reviewed and approved by the Ege University Clinical Research Ethics Committee under reference number 14–3/14. This ethical approval ensured compliance with established ethical standards and the protection of participants’ rights and well-being in accordance with the Declaration of Helsinki.

### POP analyses in blood and stomach and omental tissue samples

The study aimed to analyze 32 targeted persistent organic pollutants (POPs) in various biological matrices, including blood, cancerous and non-cancerous stomach tissues, and omental tissues from both control and cancer groups. The targeted POP compounds included: *Organochlorine pesticides (OCPs):* α-HCH, β-HCH, γ-HCH, δ-HCH, *p,p′*-DDD, *p,p′*-DDE, *p,p′*-DDT, heptachlor, endrin aldehyde, α-endosulfan, β-endosulfan, endosulfan sulfate, *Indicator polychlorinated biphenyls (PCBs):* PCB-28, PCB-101, PCB-138, PCB-153, PCB-180, *Dioxin-like (dl) PCBs:* PCB-81, PCB-77, PCB-105, PCB-114, PCB-126, PCB-156, PCB-157, PCB-167, PCB-169, PCB-189, and *Polybrominated diphenyl ethers (PBDEs):* PBDE-17, PBDE-47, PBDE-66, PBDE-100, PBDE-153.

### POP analyses in blood samples

Heparinized venous blood samples were collected from cancer and control patients before their respective surgeries, as well as from 20 healthy volunteers, and stored at -86 °C for subsequent analyses. The sample extraction and purification process followed previous studies with minor modifications [23–25]. Whole blood samples were spiked with PCB-103 and PCB-198 as internal standards. In the majority of chromatograms, PCB-103 was used as the internal standard for confirming the peak areas of the analyte peaks. However, in chromatograms where the PCB-103 peak was interfered with by another peak, PCB-198 was used instead. In cases where the latter was used, quantification was performed using the calibration curve equation obtained with the same internal standard. The samples underwent two-step extractions using a hexane:acetone (9:1, v/v) mixture, with vigorous vortex mixing for 1 min per extraction. The organic phases were combined, concentrated, and applied to glass columns packed with silica gel and anhydrous Na_2_SO_4_. After column conditioning with *n*-hexane, POPs were eluted according to previously established methods [23–25]. The eluents were concentrated to 200 μL under a gentle nitrogen stream. At the beginning of the study, during the analytical method development phase, each analyte was identified using the electron impact-gas chromatography-selected ion monitoring (EI-GC-SIM) technique. Some biological tissue and blood samples were also analyzed with the same technique before and after standard addition. However, since the EI-GC-SIM technique did not offer sufficient sensitivity for measuring POP derivatives in biological samples, we switched to the much more sensitive gas chromatography-electron capture detection (GC-ECD) technique. GC-ECD was used to quantify OCPs, PCBs, and PBDEs. Overlapping peaks between certain POP derivatives (e.g., dieldrin and PCB-77; PCB-118 and β-endosulfan; methoxychlor and PCB-156; PBDE-66 and PCB-169) necessitated the use of two distinct analytical methods, both employing an Agilent 6890 GC-ECD system with an Agilent HP-5MS capillary column (50 × 0.25 mm × 0.25 μm), as shown in the following table.

**Table:** 

Parameter	Method-1	Method-2
Injection temperature (^o^C)	200	270
Injection volume (μL)	1 (splitless)	1 (splitless)
Detector temperature (^o^C)	300	300
**Oven temperature program**		
Initial temperature (^o^C)	80	90
*a*	To 180 °C with a rate of 15 °C/min (5 min hold)	2 min hold
*b*	To 250 °C with a rate of 15 °C/min (27 min hold)	To 180 °C with a rate of 25 °C/min (2 min hold)
*c*	To 280 °C with a rate of 40 °C/min (30 min hold)	To 220 °C with a rate of 1.5 °C/min (2 min hold)
*d*	–	To 275 °C with a rate of 3 °C/min (15 min hold)
Column	50 m HP-5MS	50 m HP-5MS
Carrier gas	Nitrogen	Nitrogen
Gas pressure (psi)	50	60

Due to acidification for fat removal, certain OCP congeners (dieldrin, endrin, methoxychlor) underwent partial degradation and could not be reliably quantified [26].

### POP analyses in human tissue samples

Tissue sample extraction and purification followed previously described methodologies with minor adaptations [23–25]. Approximately 0.4 g of cancerous and non-cancerous stomach tissue and 0.2 g of omental tissue were homogenized. Internal standards (15 ng PCB-103 and 10 ng PCB-198) were added, and the solvent was evaporated under a gentle nitrogen stream. Samples were mixed with anhydrous Na₂SO₄ (1:5, w/w) and extracted using an automatic Buchi E-816 system with *n*-hexane:dichloromethane (1:1, v/v) for 2 h. Extracts were concentrated, and half was stored at −86 °C for lipid content analysis, while the other half underwent POP analysis. Lipid removal involved treatment with 2.5 mL H₂SO₄ and centrifugation. Final extracts were concentrated to 50 μL before GC-ECD analysis, following the same quantification methods as blood samples.

### Microsomal pellet ısolation from human tissues and EROD activity

Microsomal activity was assessed by homogenizing cancerous and non-cancerous stomach tissues in chilled phosphate buffer (50 mM, pH 7.4, 0.9% NaCl). Homogenates were centrifuged at 12,000 × g for 20 min at 4 °C, and supernatants were further centrifuged at 105,000 × g for 60 min at 4 °C. The resulting microsomal pellets were resuspended in 100 mM phosphate buffer (pH 7.4, 25% glycerol, 0.1% EDTA) and stored at − 86 °C until analysis. Ethoxyresorufin-O-deethylase (EROD) activity was measured fluorometrically (excitation/emission: 537/583 nm) according to Burke and Mayer [27].

### Macromolecular damage parameters in human urine samples

Levels of macromolecular damage markers, including 8-OHdG and tyrosine derivatives, were measured using high-performance liquid chromatography (HPLC) following established protocols [22, 28]. Urine samples (5 mL) were treated with 3.3 M H₂SO₄ and sodium tungstate. Analyte concentrations were determined using HPLC with UV (280 nm) and fluorescence (ex./em.: 280 nm/410 nm) detection. Chromatographic separation was performed using a Chromospher C18 column with a flow rate of 0.8 mL/min. Samples were lyophilized and analyzed by Thermo TSQ Quantiva LC–MS/MS. Urinary creatinine concentrations were determined using the Jaffé method [29].

### Genetic polymorphisms

#### DNA extraction from human lymphocyte

Blood samples (2 mL) were collected in heparinized tubes, and genomic DNA was extracted using the Thermo GeneJet Genomic DNA Purification Kit according to the manufacturer’s instructions. DNA samples were stored at − 20 °C until genotyping analysis.

#### Genotyping procedure

GSTM1 and GSTT1 deletions were detected via multiplex PCR [30]. GSTP1 I105V polymorphism was analyzed using PCR–RFLP [31]. CYP1A1 T6235C polymorphism was determined using PCR–RFLP [32]. OGG1 Ser326Cys polymorphism was identified using PCR–RFLP [33].

### Statistical analysis

Data analysis was conducted using IBM SPSS (v.25). Normality was assessed using the Shapiro–Wilk test. As data were non-parametric, the Mann–Whitney U test was used for group comparisons. Spearman correlation analysis was performed to evaluate associations between blood and tissue POP levels. Logistic regression models were employed to assess gastric cancer risk, adjusting for sex, age, and smoking status. Given the number of individual POP comparisons, the Benjamini–Hochberg false discovery rate (FDR) correction (*q* = 0.05) was applied separately to tissue and blood matrices to control for multiple testing. Aggregate (total) measures were evaluated independently and were not included in the FDR adjustment. Unless otherwise specified, a two-sided p-value < 0.05 was considered statistically significant.

## Results

### Study population

The demographic characteristics of the study population are summarized in Table S1. The control and cancer groups differed substantially with respect to age, gender distribution, and smoking prevalence. Controls were predominantly younger than 45 years, whereas most cancer patients were between 56 and 75 years of age. Smoking was more common among cancer patients. To account for these differences, all regression analyses were adjusted for age, gender, and smoking status. Alcohol consumption was low in both groups. All tumors were histologically classified as adenocarcinoma.

### Human blood POP levels

**Fig. 1:**
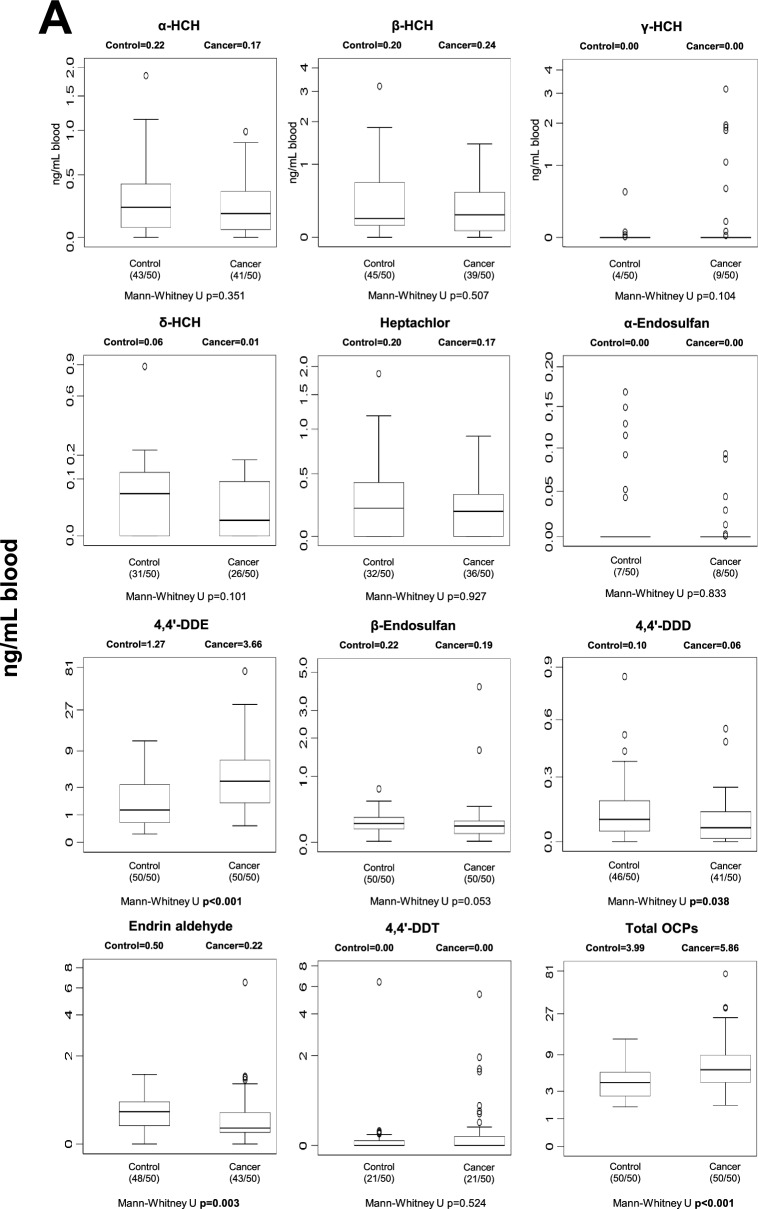

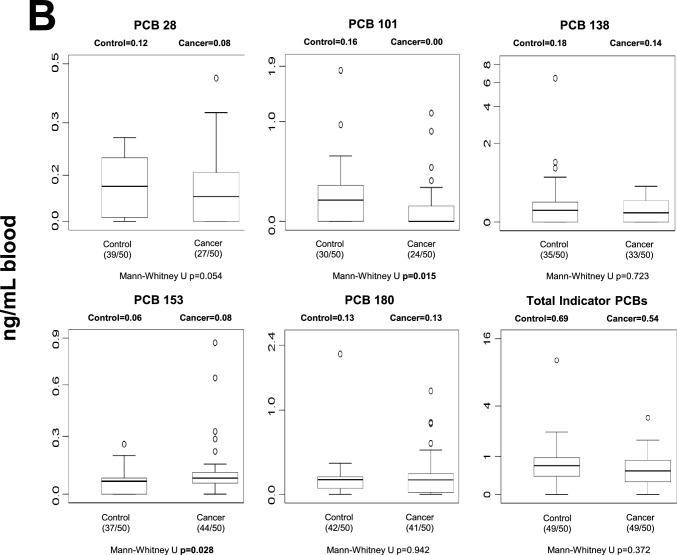

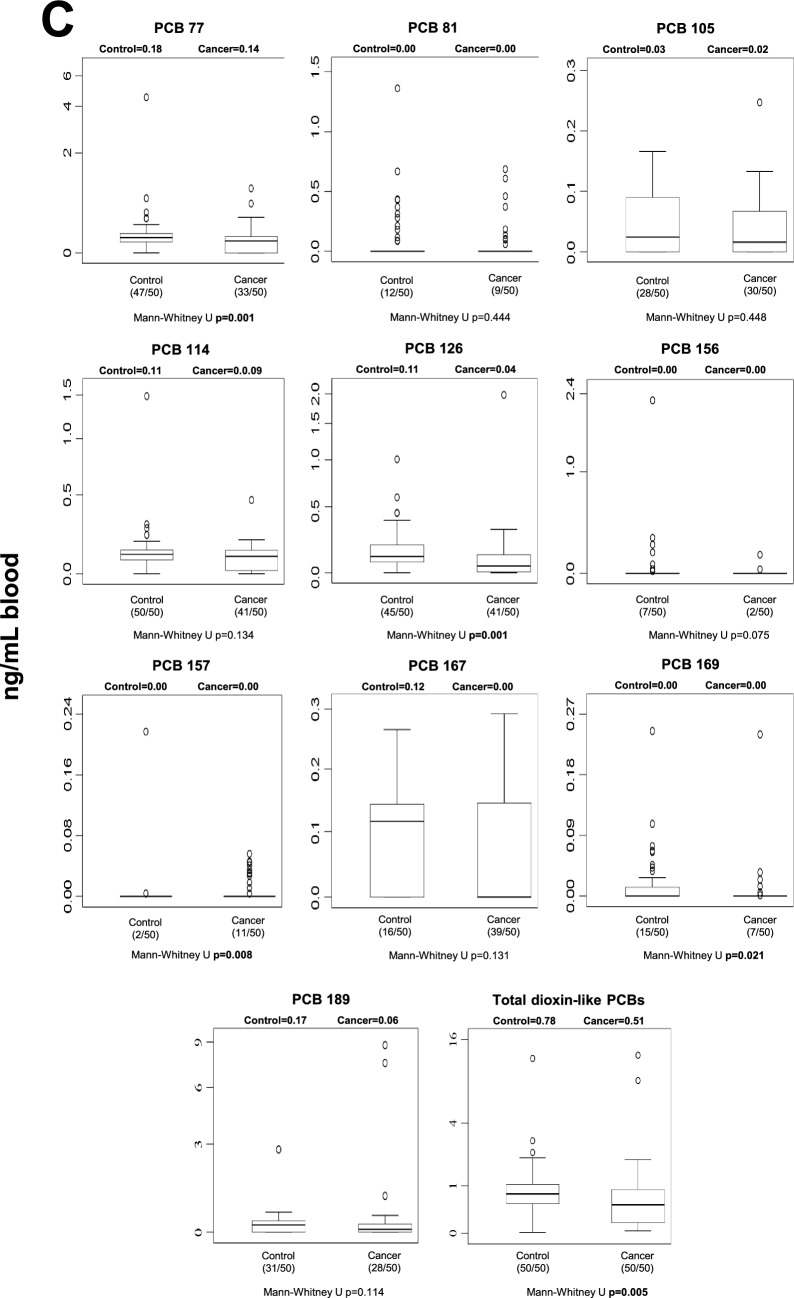

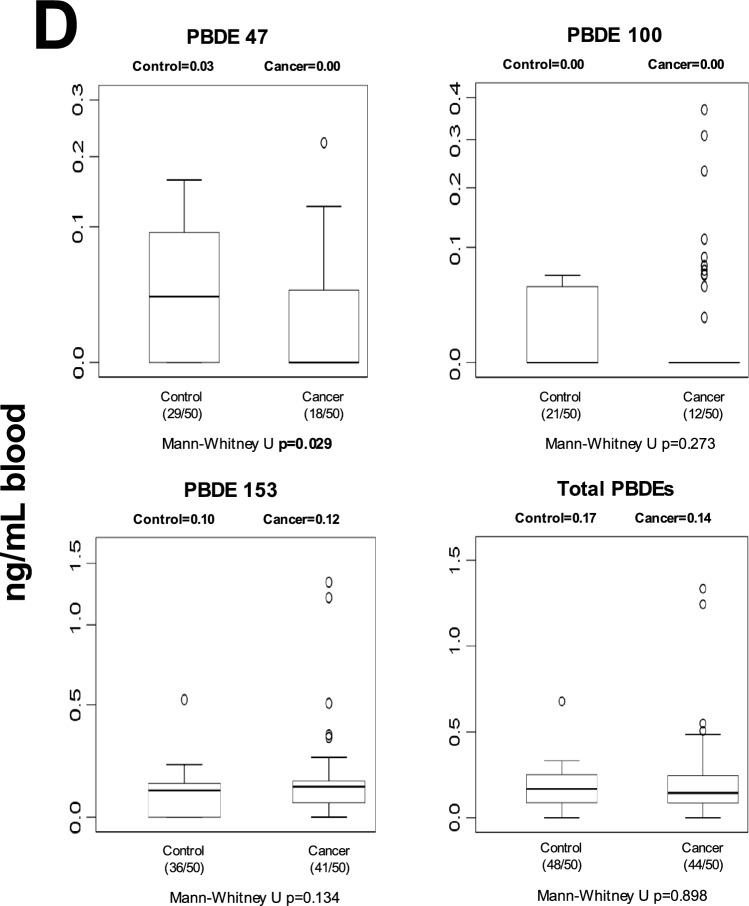
Median blood levels and distribution of POPs in control and cancer groups. Values are expressed as ng/mL whole blood. Numbers in parentheses indicate the number of individuals with detectable levels relative to the total group size. Statistical significance shown in the figure represents unadjusted comparisons; associations that remained significant after Benjamini–Hochberg FDR correction are described in the Results text. **A** blood OCP levels; **B** blood non-dioxin-like PCB levels; **C** blood dioxin-like PCB levels; **D** blood PBDE levels

Blood POP concentrations differed between control and cancer groups across several chemical classes (Fig. 1). After Benjamini–Hochberg false discovery rate (FDR) correction (*q* = 0.05), five individual POPs remained significantly different between groups: p,p′-DDE, PCB-77, PCB-126, Endrin aldehyde, and PCB-157 (Table S2). In addition, total OCPs and total dioxin-like PCBs also remained statistically significant after correction. In general, selected OCPs and specific PCB congeners were elevated in cancer patients, whereas some compounds showed higher levels in controls, indicating compound-specific differences rather than a uniform increase across all POP classes. Nominally significant differences that did not withstand FDR correction are shown in Fig. 1 and should be interpreted cautiously.

### Human tissue POP levels

#### Stomach tissue

**Fig. 2:**
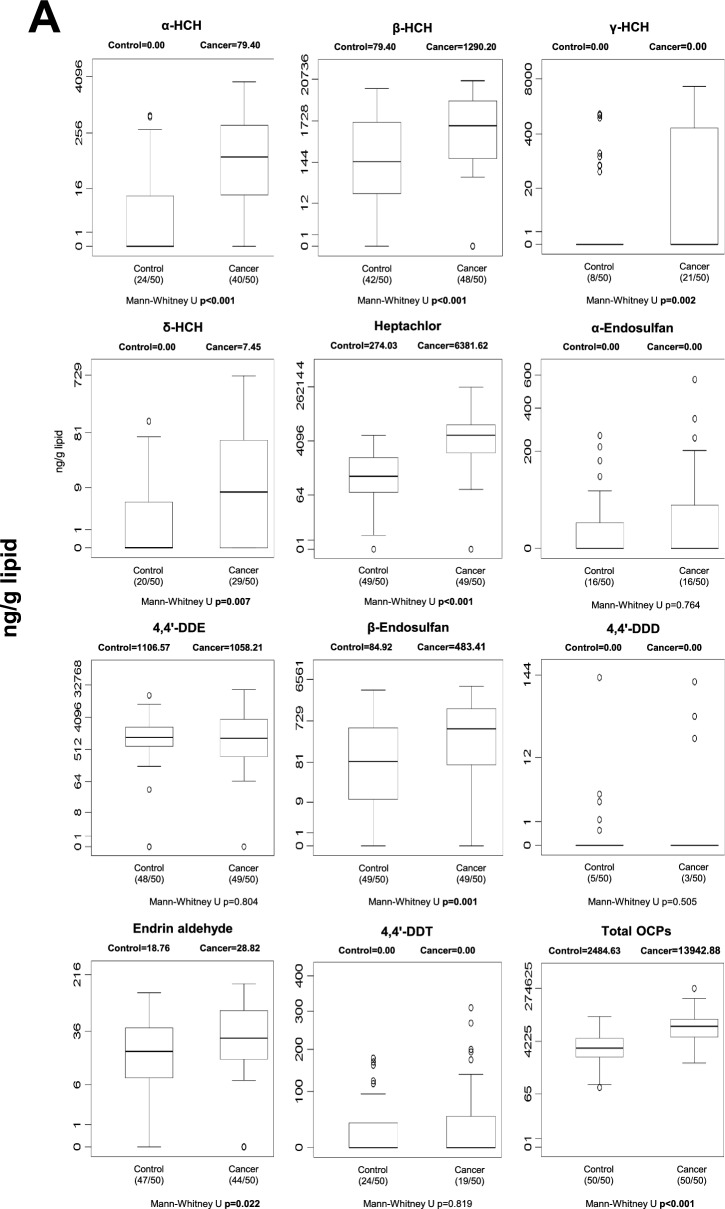

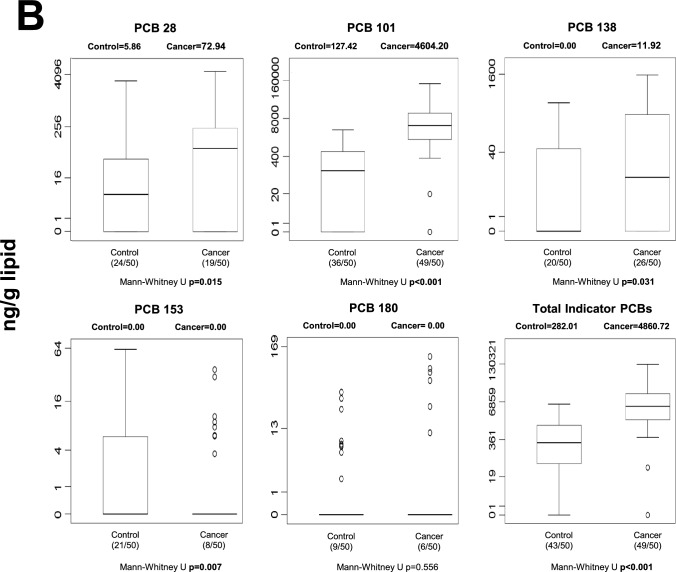

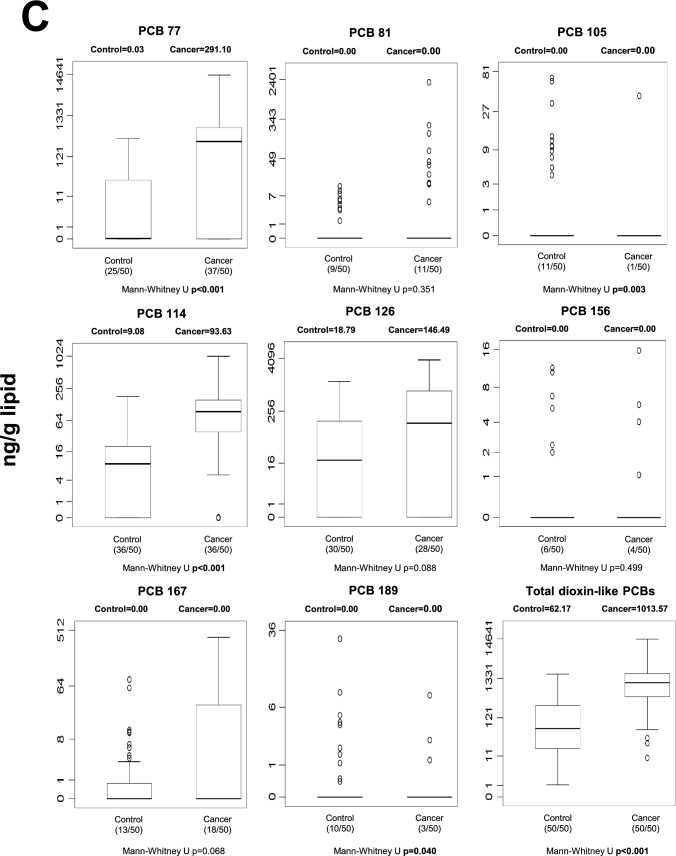

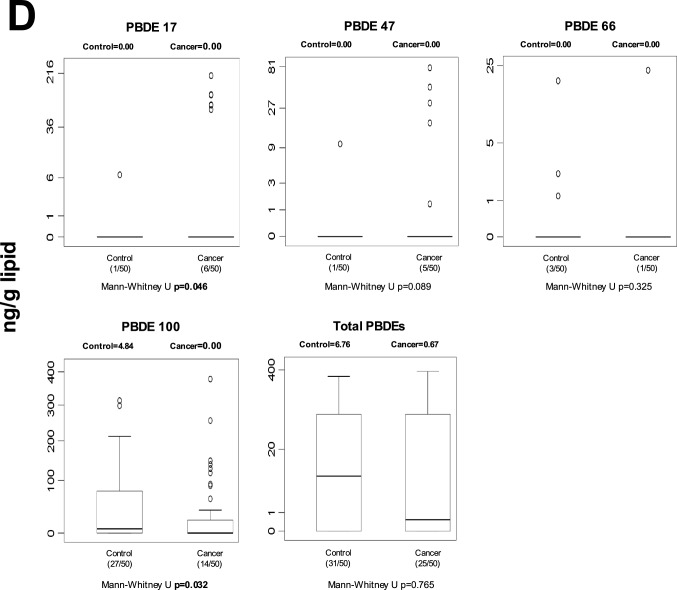
Median gastric tissue levels and distribution of individual and total POPs in control and cancer groups. Values are expressed as ng/g lipid. Numbers in parentheses indicate detectable samples relative to total group size. Unadjusted p-values are displayed in the figure; FDR-adjusted results are described in the Results section. **A** stomach tissue OCP levels; **B** stomach tissue non-dioxin-like PCB levels; **C** stomach tissue dioxin-like PCB levels; **D** stomach tissue PBDE levels

Marked differences in POP concentrations were observed in gastric tissue (Fig. 2). Following FDR correction, 13 individual POPs remained significantly different between cancer and control tissues (Table S2). These included multiple HCH isomers (α-, β-, γ-, and δ-HCH), heptachlor, β-endosulfan, endrin aldehyde, and several PCB congeners (PCB-28, -77, -101, -105, -114, and -153). At the class level, total OCPs and total dioxin-like PCBs also remained statistically significant after correction. In general, concentrations of these compounds were higher in cancerous gastric tissue compared to controls, suggesting robust tissue-specific accumulation patterns associated with gastric cancer. Unadjusted p-values are displayed in Fig. 2; FDR-adjusted results are described in the text.

#### Omental adipose tissue

In omental adipose tissue, several POPs differed between groups (Figure S1). After FDR correction, four individual POPs remained significantly different: PBDE-153, PCB-153, PCB-180, and PCB-28 (Table S2). At the chemical class level, total OCPs, total indicator PCBs, total dioxin-like PCBs, and total PBDEs also retained statistical significance. These findings indicate consistent accumulation of selected POP classes in adipose tissue of cancer patients.

### Cross-compartment correlations

Correlations between blood and tissue POP levels are presented in Figure S2 and Table S3. Although numerous nominal correlations were observed, only a subset remained statistically significant after FDR correction. After correction: Six correlations remained significant in blood–tumor comparisons, two in blood–omentum comparisons, and six in tumor–omentum comparisons. Significant correlations were most consistently observed for selected OCPs (particularly β-HCH, δ-HCH, and 4,4′-DDE) as well as specific PCB and PBDE congeners. These findings suggest that certain POPs demonstrate stable cross-compartment distribution patterns, whereas others show more variable associations.

### Gastric tissue CYP1A activities

CYP1A activities did not significantly differ between non-cancerous and cancerous gastric tissues (data not shown). Although several nominal correlations between CYP1A activity and individual POPs were observed (Fig S3), none remained statistically significant after FDR correction (data not shown). These results indicate that measured CYP1A activity was not robustly associated with tissue POP levels in this cohort.

### Urinary oxidative damage biomarkers

**Fig. 3:**
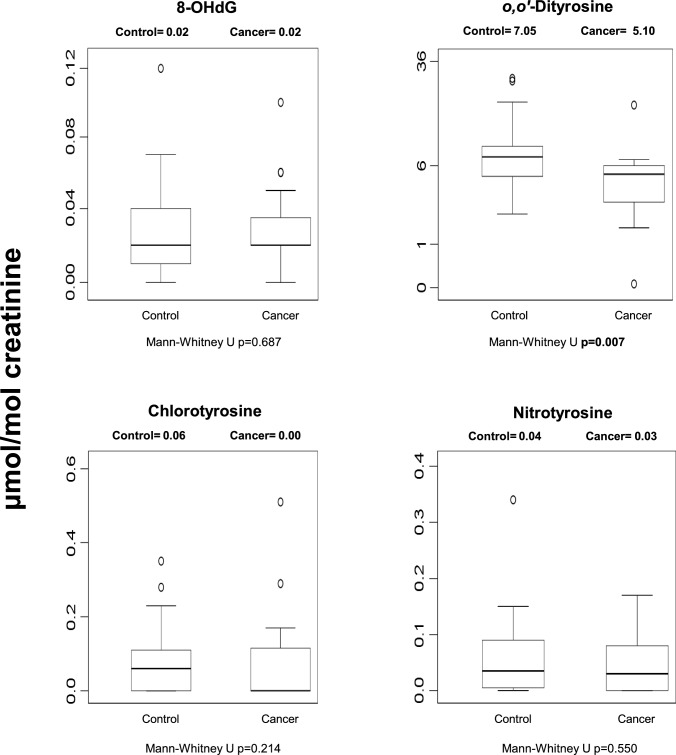
Median urinary oxidative damage biomarkers in control and cancer groups. Values are expressed as μmol/mol creatinine. Statistical significance displayed in the figure represents unadjusted comparisons; FDR-adjusted results are reported in the Results text

Among four urinary oxidative damage biomarkers (Fig. 3), only o,o′-dityrosine differed significantly between groups after FDR correction (FDR-adjusted *p* = 0.028), with lower levels observed in cancer patients. No significant differences were observed for 8-OHdG, chlorotyrosine, or nitrotyrosine following correction. Nominally significant findings that did not withstand adjustment are shown in Fig. 3.

### Genetic polymorphism and gastric cancer risk

Genotype distributions and corresponding odds ratios are shown in Table 1. In crude analyses, none of the investigated polymorphisms demonstrated a statistically robust association with gastric cancer risk. The CYP1A1 variant genotype showed a borderline association in crude analysis (OR = 2.25, 95% CI: 0.989–5.121; *p* = 0.049), which was attenuated after adjustment for age, sex, and smoking status. For GSTP1, the crude association was not statistically significant (OR = 1.47, 95% CI: 0.690–3.125; *p* = 0.319). In the multivariable model adjusted for age, sex, and smoking, the point estimate increased (adjusted OR = 5.21, 95% CI: 0.973–27.869; *p* = 0.0499). However, the confidence interval was wide and approached unity, indicating limited precision of the estimate.

**Table 1:** Association between genetic polymorphisms and gastric cancer risk

Polymorphism	Crude OR (95% CI)	P-value	Adjusted OR (95% CI)*	P-value
CYP1A1 (Variant H + M)	2.250 (0.989–5.121)	0.049	1.347 (0.261–6.947)	0.722
OGG1 (Variant)	1.506 (0.715–3.173)	0.282	0.855 (0.200–3.653)	0.833
GSTP1 (Variant)	1.469 (0.690–3.125)	0.319	5.208 (0.973–27.869)	0.049
GSTM1 (Null)	1.477 (0.712–3.062)	0.295	0.663 (0.159–2.763)	0.572
GSTT1 (Null)	1.426 (0.615–3.306)	0.408	3.521 (0.622–19.927)	0.155

^*^Adjusted for age, gender, and smoking status

No statistically significant associations were observed for GSTM1, GSTT1, or OGG1 in either crude or adjusted models. Stratified analyses were subsequently performed (Table S4). After FDR correction, statistically significant associations were observed in age-stratified models for all investigated polymorphisms, whereas no significant associations remained in gender- or smoking-stratified analyses. These findings suggest potential effect modification by age, although subgroup results should be interpreted cautiously due to limited sample size.

## Discussion

This study investigated the distribution of persistent organic pollutants (POPs) across blood, gastric tissue, and omental adipose tissue in relation to gastric cancer, while also examining biotransformation enzyme activity, oxidative damage markers, and genetic susceptibility. To our knowledge, this is the first study to quantify a broad panel of POP derivatives in both non-cancerous and malignant gastric tissues within the same population. The present work represents the final component of a broader project evaluating POP accumulation in other malignancies, including breast cancer [7] and kidney cancer [8], where strong associations between tissue POP levels and carcinogenesis were observed.

Although the POP derivatives and biological matrices do not completely overlap, two studies reporting comparable tissue POP levels are available. Perrot-Applanat and coworkers [16] measured OCP, PCB, and PBDE derivatives—also analyzed in our study—in omentum tissue from gastric cancer and noncancer patients. Overall, OCP concentrations were within the same order of magnitude as our findings; however, the five OCPs quantified in their study tended to be moderately higher than those measured in our samples (Tables S5A–B). For PCBs, approximately half of the 15 congeners were reported at levels 100–1000-fold lower than ours, whereas PCB 156, 157, 167, 169, and 189 were comparable (Tables S5C–D). Notably, while OCP and PBDE levels were expressed in ng/g lipid weight, PCB concentrations were reported in pg/g lipid weight, raising the possibility of reporting inconsistencies. Alternatively, geographic, environmental, occupational, or lifestyle-related factors may explain the observed differences. The earlier study by Wasserman et al. (1978) [34] reported DDE, DDD, and DDT levels in cancer and noncancer tissues; however, DDD and DDT concentrations were several 100-fold higher than those observed in both our study and that of Perrot-Applanat (Tables S5A–B), for reasons that remain unclear. PCB congeners were not individually coded at that time, preventing direct comparison. Overall, with the exception of the markedly elevated OCP values reported by Wasserman et al. and the moderately higher OCP levels in Perrot-Applanat’s study, tissue POP concentrations appear broadly comparable across studies.

The most consistent and statistically robust findings emerged from gastric tissue analyses. After false discovery rate (FDR) correction, 13 individual POPs remained significantly different between cancerous and non-cancerous gastric tissues, together with total OCPs and total dioxin-like PCBs (Table S2). Several HCH isomers and multiple PCB congeners were elevated in malignant tissue, including compounds classified as carcinogenic or possibly carcinogenic by the International Agency for Research on Cancer (IARC) [13].

The persistence of these associations after correction for multiple testing strengthens the credibility of the observed differences and reduces the likelihood that they represent chance findings. Rather than suggesting uniform elevation across all POP classes, the data indicate selective enrichment of specific compounds in tumor tissue. This pattern is consistent with the lipophilic properties and long biological half-lives of these chemicals, which facilitate accumulation in lipid-rich cellular compartments. While causality cannot be inferred from this cross-sectional design, the findings are compatible with a potential role of tissue-level POP accumulation in gastric carcinogenesis. Cross-compartment analyses further supported biologically coherent distribution patterns. After FDR correction, six significant correlations persisted in blood–tumor comparisons, two in blood–omentum comparisons, and six in tumor–omentum comparisons (Table S3). Notably, several compounds demonstrated consistent correlations across more than one compartment pairing. Significant associations were particularly observed for selected HCH isomers (β-HCH and δ-HCH), 4,4′-DDE, β-endosulfan, PCB 126, PCB 138, PCB 167, and PBDE 100 across blood–tumor and tumor–omentum comparisons. The recurrence of correlations for the same compounds across blood–tumor, blood–omentum, and tumor–omentum comparisons suggests relatively stable cross-compartment distribution patterns for specific POPs. In particular, the presence of multiple significant blood–tumor correlations indicates that, for selected compounds, circulating levels may reflect tissue burden to a meaningful extent. The reproducibility of associations for compounds such as 4,4′-DDE supports the hypothesis of relatively stable equilibrium dynamics between circulation and lipid-rich tissues, consistent with previous observations in other cancer contexts. For example, Artacho-Cordon et al. [35] reported positive correlations between serum and adipose tissue 4,4′-DDE levels in breast cancer patients. The use of blood as a surrogate for tissue POP burden has also been previously explored in wildlife studies [25]. However, these relationships were compound-specific rather than universal. While several correlations remained statistically significant after FDR correction, not all POPs demonstrated consistent cross-compartment concordance. Therefore, although our findings support the potential utility of blood measurements as a proxy for tissue accumulation for selected POPs, the exploratory nature of these analyses and the modest sample size warrant cautious interpretation. Larger studies are needed to confirm the robustness and generalizability of these cross-compartment relationships.

In omental adipose tissue, four individual POPs and all major chemical classes remained significantly different after FDR correction (Table S2). These findings reinforce the concept of adipose tissue as a long-term storage compartment for lipophilic pollutants. Adipose depots are known to accumulate POPs over time and may release them under conditions such as weight loss [36]. The control group in the present study consisted of patients undergoing stomach reduction surgery, a procedure frequently associated with significant weight loss and altered nutrient absorption [37], which may influence mobilization dynamics. Given the anatomical proximity of omental fat to gastric tissue, regional redistribution could contribute to localized exposure, although temporal relationships cannot be established within the current design. In blood, five individual POPs and selected chemical classes remained statistically significant after FDR correction. Some of these compounds have been classified as carcinogenic or possibly carcinogenic by IARC [13]. Nevertheless, the more limited number of associations compared with gastric tissue suggests that circulating levels may not fully capture tissue-specific accumulation patterns. Blood measurements may therefore provide partial, but not comprehensive, insight into internal POP distribution.

CYP1A enzymes play a central role in xenobiotic metabolism, including the biotransformation of certain POPs. Despite this biological relevance, CYP1A activity did not differ between cancerous and non-cancerous gastric tissues, and no associations with individual POPs remained significant after FDR correction. These findings suggest that CYP1A activity may not be strongly determined by single-compound exposure levels but could reflect cumulative mixture effects or broader regulatory mechanisms.

Among oxidative damage markers, only urinary *o,o′*-dityrosine remained significantly different between groups after FDR correction, with lower levels observed in cancer patients. Oxidized tyrosine residues are known to be cleaved and excreted in urine as part of protein damage processing pathways. Reduced urinary excretion may therefore reflect altered oxidative stress handling or differences in protein turnover dynamics. Given the limited biomarker panel and cross-sectional design, these findings should be interpreted as hypothesis-generating.

Polymorphisms in genes encoding biotransformation enzymes (CYP1A1, GSTP1, GSTM1, GSTT1) and the DNA repair enzyme OGG1 were evaluated for their association with gastric cancer risk. In crude analysis, CYP1A1 variant carriers showed an increased risk estimate, consistent with some previous reports [38], although other studies have reported null findings [39, 40]. After adjustment, this association was attenuated. For GSTP1, the adjusted model yielded an elevated odds ratio (OR = 5.21), but the wide confidence interval (95% CI: 0.973–27.869) and borderline p-value (0.0499) indicate limited precision. Previous literature on GSTP1 and gastric cancer risk has been inconsistent, with some studies suggesting possible associations [41] and others reporting no significant relationship [42, 43]. No statistically robust associations were observed for GSTM1, GSTT1, or OGG1 in overall models. Age-stratified analyses suggested potential effect modification, as age-stratified models remained statistically significant after FDR correction, whereas gender- and smoking-stratified models did not. However, given the modest subgroup sizes and wide confidence intervals, these findings require confirmation in larger cohorts and should be interpreted cautiously.

Strengths of this study include multi-compartment POP measurement, integration of biochemical and genetic susceptibility markers, and rigorous control for confounding and multiple testing using the Benjamini–Hochberg FDR procedure. The consistency of gastric tissue findings after correction enhances confidence in the robustness of the principal observations. Limitations include the cross-sectional design, modest sample size, and limited power for subgroup genetic analyses. The observational nature of the study precludes inference regarding causality or temporal directionality. An additional limitation concerns potential imbalance in baseline characteristics between cases and controls, including differences in age and sex distribution, which may have introduced residual confounding despite statistical adjustment. Although multivariable models accounted for major covariates, the relatively modest sample size limited the ability to control for a broader range of potential confounders simultaneously. The sample size may also have reduced statistical power, particularly in subgroup and genetic analyses, contributing to wide confidence intervals and limited precision of some estimates. Accordingly, the findings of this study should be interpreted as exploratory and hypothesis-generating rather than definitive. While the observed tissue-level associations were consistent after multiple comparison correction, confirmation in larger, well-powered, and prospectively designed studies is necessary before firm causal inferences can be drawn.

Taken together, the findings are consistent with a multifactorial framework in which selective accumulation of specific POPs in gastric tissue may be associated with gastric cancer, while host genetic variability could potentially modulate susceptibility. The strongest and most reproducible signals were observed at the tissue level. Prospective and mechanistic studies are required to clarify causal pathways and to determine whether specific genetic profiles modify risk in exposed populations.

## Supplementary Information

Below is the link to the electronic supplementary material.

Supplementary file1 (DOCX 646 KB)

## Data Availability

Data are available from the corresponding author upon reasonable request.

## Abbreviations

CYP1A1: Cytochrome P450 1A1
EROD: Ethoxyresorufin-O-deethylase
GC-ECD: Gas chromatography-electron capture detection
EI-GC-SIM: Electron impact-gas chromatography-selected ion monitoring
FDR: False discovery rate
GSTM1: Glutathione S-transferase M1
GSTP1: Glutathione S-transferase P1
GSTT1: Glutathione S-transferase T1
8-OHdG: 8-Hydroxy-2′-deoxyguanosine
hOGG1: Human 8-oxoguanine glycosylase 1
IARC: International Agency for Research on Cancer
OCPs: Organochlorine pesticides
ORs: Odds ratios
PBDEs: Polybromodiphenyl ethers
PCBs: Polychlorinated biphenyls
POPs: Persistent organic pollutants
