# Supplementary material for: Tissue levels of persistent organic pollutants and biotransformation enzyme polymorphisms in human stomach cancer
Source: Cancer Causes Control. 2026 Mar 24;37(4):68. doi: 10.1007/s10552-026-02154-z (PMC13013339; doi:10.1007/s10552-026-02154-z)
Supplement: Supplementary file 1 — Supplementary file1 (DOCX 646 KB) [file 10552_2026_2154_MOESM1_ESM.docx]

**Supplementary Information, Kocagöz et al.**

**Table S1** Demographics and Clinical Characteristics of Control, Cancer, and Healthy Volunteer Groups

| **Demographics and Clinical Characteristics** | **Control**  **(n=50) n(%)** | **Cancer**  **(n=50) n(%)** | **Healthy Volunteers**  **(n=20) n(%)** |
| --- | --- | --- | --- |
| **Gender** | | |  |
| Women | 34 (68) | 14 (28) | 10 (50) |
| Men | 16 (32) | 36 (72) | 10 (50) |
| **Age (Year)** | | |  |
| ≤45 | 33 (66) | 2 (4) | 18 (90) |
| 46-55 | 11 (22) | 6 (12) | 1 (5) |
| 56-65 | 6 (12) | 14 (28) | 1 (5) |
| 66-75 | - | 19 (38) | - |
| >75 | - | 9 (18) | - |
| **Smoking** | | |  |
| Yes | 8 (16) | 24 (48) | 10 (50) |
| 1-5/per day | - | 1 (2) | 4 (20) |
| 5-10/ per day | - | 1 (2) | 1 (5) |
| 15-40/ per day | 8 (16) | 20 (40) | 4 (20) |
| rarely | - | - | 1 (5) |
| Quit before surgery | - | 2 (4) | - |
| No | 42 (84) | 26 (52) | 10 (50) |
| **Alcohol (glasses per day)** | | |  |
| Yes | 1 (2) | 5 (10) | 9 (45) |
| 1< | 1 (2) | 3 (6) | 9 (45) |
| 1 | - | - | - |
| 1> | - | 2 (4) | - |
| No | 49 (98) | 45 (90) | 11 (55) |
| **Chronic disease** | | |  |
| Yes | 10 (20) | 19 (38) | 4 (20) |
| No | 40 (80) | 31 (62) | 16 (80) |
| **Drug usage** | | |  |
| Yes | 10 (20) | 19 (38) | 3 (15) |
| No | 40 (80) | 31 (62) | 17 (85) |
| **Surgery type** | | |  |
| Laparoscopic Cholecystectomy | 1 (2) | - | - |
| Laparoscopic Sleeve Gastrectomy | 48 (96) | - | - |
| Total Gastrectomy | 1 (2) | 5 (10) | - |
| Total Gastrectomy and D2 Lymph Node dissection | - | 31 (62) | - |
| Distal Subtotal Gastrectomy and D2 Lymph Node dissection | - | 10 (20) | - |
| Distal Subtotal Gastrectomy | - | 2 (4) | - |
| Other | - | 2 (4)**^a^** | - |
| **Tumor Type** | | |  |
| Adenocarcinoma | - | 50 (100) | - |

**^a^**: D2 Lymph Node dissection, distal pancreatectomy and splenectomy; Total Gastrectomy, D2 Lymph Nodule dissection, splenectomy and right ovarianectomy.

**Table S2** False discovery rate (FDR) correction for individual POP comparisons in blood, stomach tissue and omentum tissue samples

| **POP** | **Crude p-value** | **FDR-adjusted p-value (BH)** | **Significant after FDR (q < 0.05)** |
| --- | --- | --- | --- |
| ***BLOOD*** | | | |
| p,p′-DDE | <0.001 | 0.009 | **Yes** |
| PCB-77 | 0.001 | 0.009 | **Yes** |
| PCB-126 | 0.001 | 0.009 | **Yes** |
| Endrin aldehyde | 0.003 | 0.021 | **Yes** |
| PCB-157 | 0.008 | 0.045 | **Yes** |
| PCB-101 | <0.015 | 0.070 | No |
| PCB-169 | 0.021 | 0.078 | No |
| PCB-153 | 0.028 | 0.092 | No |
| PBDE-47 | 0.029 | 0.092 | No |
| *p,p′-*DDD | 0.038 | 0.108 | No |
| *β*-Endosulfan | 0.053 | 0.135 | No |
| PCB-28 | 0.054 | 0.135 | No |
| *δ-*HCH | 0.101 | 0.212 | No |
| *γ*-HCH | 0.104 | 0.212 | No |
| PCB-167 | 0.131 | 0.224 | No |
| PCB-114 | 0.134 | 0.224 | No |
| PBDE-153 | 0.134 | 0.224 | No |
| PBDE-100 | 0.273 | 0.425 | No |
| *α*-HCH | 0.351 | 0.525 | No |
| PCB-81 | 0.444 | 0.622 | No |
| PCB-105 | 0.448 | 0.622 | No |
| *β*-HCH | 0.507 | 0.677 | No |
| *p,p′-*DDT | 0.524 | 0.677 | No |
| PCB-138 | 0.723 | 0.869 | No |
| *α-*Endosulfan | 0.833 | 0.942 | No |
| Heptachlor | 0.927 | 0.982 | No |
| PCB-180 | 0.942 | 0.982 | No |
| TOTAL OCPs | <0.001 | 0.004 | **Yes** |
| TOTAL indicator PCBs | 0.372 | 0.496 | No |
| TOTAL dioxin-like PCBs | 0.005 | 0.010 | **Yes** |
| TOTAL PBDEs | 0.898 | 0.898 | No |
| ***TUMOR TISSUE*** | | | |
| *α*-HCH | <0.001 | 0.0029 | **Yes** |
| *β*-HCH | <0.001 | 0.0029 | **Yes** |
| Heptachlor | <0.001 | 0.0029 | **Yes** |
| PCB-101 | <0.001 | 0.0029 | **Yes** |
| PCB-77 | <0.001 | 0.0029 | **Yes** |
| PCB-114 | <0.001 | 0.0029 | **Yes** |
| *β*-Endosulfan | 0.001 | 0.0041 | **Yes** |
| γ-HCH | 0.002 | 0.0058 | **Yes** |
| PCB-105 | 0.003 | 0.0087 | **Yes** |
| *δ*-HCH | 0.007 | 0.0145 | **Yes** |
| PCB-153 | 0.007 | 0.0145 | **Yes** |
| PCB-28 | 0.015 | 0.0242 | **Yes** |
| Endrin aldehyde | 0.022 | 0.0390 | **Yes** |
| PCB-138 | 0.031 | 0.0517 | No |
| PCB-189 | 0.040 | 0.0622 | No |
| PBDE-100 | 0.032 | 0.0536 | No |
| PBDE-17 | 0.046 | 0.0711 | No |
| PCB-167 | 0.068 | 0.103 | No |
| PCB-126 | 0.088 | 0.128 | No |
| PBDE-47 | 0.089 | 0.128 | No |
| PCB-81 | 0.351 | 0.457 | No |
| PBDE-66 | 0.325 | 0.457 | No |
| PCB-156 | 0.499 | 0.603 | No |
| *p,p′-*DDD | 0.505 | 0.603 | No |
| PCB-180 | 0.556 | 0.645 | No |
| *α*-Endosulfan | 0.764 | 0.853 | No |
| *p,p′-*DDE | 0.804 | 0.886 | No |
| *p,p′-*DDT | 0.819 | 0.886 | No |
| TOTAL OCPs | <0.001 | 0.004 | **Yes** |
| TOTAL indicator PCBs | <0.001 | 0.004 | **Yes** |
| TOTAL dioxin-like PCBs | <0.001 | 0.004 | **Yes** |
| TOTAL PBDEs | 0.765 | 0.765 | No |
| ***OMENTUM TISSUE*** | | | |
| PCB-153 | 0.005 | 0.029 | **Yes** |
| PCB-180 | 0.005 | 0.029 | **Yes** |
| PCB-28 | 0.008 | 0.037 | **Yes** |
| PBDE-153 | 0.001 | 0.029 | **Yes** |
| *δ*-HCH | 0.004 | 0.058 | No |
| *p,p′-*DDE | 0.012 | 0.058 | No |
| PCB-167 | 0.033 | 0.137 | No |
| PCB-126 | 0.049 | 0.177 | No |
| Endrin aldehyde | 0.056 | 0.181 | No |
| *β*-HCH | 0.997 | 0.997 | No |
| *p,p′-*DDD | 0.126 | 0.261 | No |
| *p,p′-*DDT | 0.543 | 0.720 | No |
| Heptachlor | 0.506 | 0.704 | No |
| *α*-Endosulfan | 0.758 | 0.880 | No |
| Endosulfan.SO_4_ | 0.728 | 0.880 | No |
| *β*-Endosulfan | 0.111 | 0.247 | No |
| PCB-101 | 0.138 | 0.276 | No |
| PCB-138 | 0.158 | 0.293 | No |
| PCB-77 | 0.080 | 0.160 | No |
| PCB-105 | 0.106 | 0.247 | No |
| PCB-114 | 0.762 | 0.880 | No |
| PCB-156 | 0.120 | 0.255 | No |
| PCB-169 | 0.098 | 0.228 | No |
| PCB-189 | 0.225 | 0.368 | No |
| PBDE-17 | 0.160 | 0.293 | No |
| PBDE-47 | 0.780 | 0.880 | No |
| PBDE-66 | 0.930 | 0.997 | No |
| PBDE-100 | 0.351 | 0.528 | No |
| TOTAL OCPs | 0.046 | 0.046 | **Yes** |
| TOTAL indicator PCBs | 0.005 | 0.010 | **Yes** |
| TOTAL dioxin-like PCBs | 0.005 | 0.010 | **Yes** |
| TOTAL PBDEs | 0.033 | 0.044 | **Yes** |

*Benjamini–Hochberg false discovery rate (FDR) correction (q = 0.05) was applied separately to blood, stomach tumor tissue and omentum tissue POP comparisons. P-values reported as below a threshold (e.g., <0.001 or <0.015) were conservatively treated as 0.001 and 0.015 in the FDR procedure.*

**Table S3** Benjamini–Hochberg false discovery rate (FDR)-adjusted p-values for cross-compartment correlations of persistent organic pollutant (POP) derivatives between blood, tumor tissue, and omental adipose tissue.

**A. Blood-Tumor**

| **POP** | **Crude**  **p-value** | **FDR-adjusted p-value (BH)** | **Significant after FDR (q < 0.05)** |
| --- | --- | --- | --- |
| α-HCH | 0.628 | 0.714 | No |
| β-HCH | 0.014 | 0.042 | **Yes** |
| γ-HCH | 0.488 | 0.714 | No |
| δ-HCH | 0.001 | 0.014 | **Yes** |
| Heptachlor | 0.306 | 0.461 | No |
| α-endosulfan | 0.187 | 0.298 | No |
| 4,4'-DDE | 0.006 | 0.035 | **Yes** |
| β-endosulfan | 0.468 | 0.714 | No |
| 4,4'-DDD | 0.144 | 0.256 | No |
| Endrin aldehit | 0.008 | 0.042 | **Yes** |
| Endosülfan sülfat | 0.091 | 0.178 | No |
| 4,4'-DDT | 0.705 | 0.805 | No |
| PCB 28 | 0.597 | 0.714 | No |
| PCB 101 | 0.301 | 0.461 | No |
| PCB 138 | 0.003 | 0.021 | **Yes** |
| PCB 153 | 0.405 | 0.617 | No |
| PCB 180 | 0.073 | 0.156 | No |
| PCB 81 | 0.885 | 0.885 | No |
| PCB 77 | 0.943 | 0.943 | No |
| PCB 114 | 0.374 | 0.617 | No |
| PCB 105 | 0.778 | 0.885 | No |
| PCB 126 | 0.112 | 0.200 | No |
| PCB 167 | 0.001 | 0.014 | **Yes** |
| PCB 156 | 0.678 | 0.805 | No |
| PCB 169 | 0.717 | 0.805 | No |
| PCB 189 | 0.038 | 0.081 | No |
| PBDE 47 | 0.670 | 0.805 | No |
| PBDE 100 | 0.348 | 0.617 | No |
| PBDE 153 | 0.864 | 0.885 | No |

**B. Blood-Omentum**

| **POP** | **Crude p-value** | **FDR-adjusted p-value (BH)** | **Significant after FDR (q < 0.05)** |
| --- | --- | --- | --- |
| α-HCH | 0.784 | 0.812 | No |
| β-HCH | 0.110 | 0.168 | No |
| γ-HCH | 0.646 | 0.726 | No |
| δ-HCH | 0.018 | 0.071 | No |
| Heptachlor | 0.081 | 0.156 | No |
| α-Endosulfan | 0.719 | 0.726 | No |
| 4,4'-DDE | 0.001 | 0.006 | **Yes** |
| β-Endosulfan | 0.678 | 0.726 | No |
| 4,4'-DDD | 0.035 | 0.123 | No |
| Endrin aldehyde | 0.762 | 0.776 | No |
| Endosulfan.SO_4_ | 0.498 | 0.626 | No |
| 4,4'-DDT | 0.827 | 0.827 | No |
| PCB 28 | 0.247 | 0.332 | No |
| PCB 101 | 0.329 | 0.394 | No |
| PCB 138 | 0.719 | 0.726 | No |
| PCB 153 | 0.035 | 0.123 | No |
| PCB 180 | 0.653 | 0.726 | No |
| PCB 77 | 0.404 | 0.518 | No |
| PCB 114 | 0.879 | 0.879 | No |
| PCB 105 | 0.946 | 0.946 | No |
| PCB 126 | 0.050 | 0.158 | No |
| PCB 167 | 0.118 | 0.168 | No |
| PCB 156 | 0.620 | 0.726 | No |
| PCB 157 | 0.604 | 0.726 | No |
| PCB 169 | 0.117 | 0.168 | No |
| PCB 189 | 0.573 | 0.726 | No |
| PBDE 47 | 0.542 | 0.726 | No |
| PBDE 100 | 0.001 | 0.006 | **Yes** |
| PBDE 153 | 0.033 | 0.123 | No |

**C. Tumor-Omentum**

| **POP** | **Crude p-value** | **FDR-adjusted p-value (BH)** | **Significant after FDR (q < 0.05)** |
| --- | --- | --- | --- |
| α-HCH | 0.319 | 0.408 | No |
| β-HCH | 0.001 | 0.006 | **Yes** |
| γ-HCH | 0.423 | 0.446 | No |
| δ-HCH | 0.011 | 0.048 | **Yes** |
| Heptachor | 0.782 | 0.782 | No |
| α-Endosulfan | 0.024 | 0.089 | No |
| 4,4'-DDE | 0.005 | 0.028 | **Yes** |
| β-Endosulfan | 0.001 | 0.006 | **Yes** |
| 4,4'-DDD | 0.917 | 0.917 | No |
| Endrin aldehyde | 0.272 | 0.408 | No |
| Endosulfan.SO_4_ | 0.924 | 0.924 | No |
| 4,4'-DDT | 0.282 | 0.408 | No |
| PCB 28 | 0.518 | 0.626 | No |
| PCB 101 | 0.836 | 0.836 | No |
| PCB 138 | 0.782 | 0.782 | No |
| PCB 153 | 0.018 | 0.089 | No |
| PCB 180 | 0.827 | 0.836 | No |
| PCB 77 | 0.548 | 0.626 | No |
| PCB 114 | 0.555 | 0.626 | No |
| PCB 105 | 0.527 | 0.626 | No |
| PCB 126 | 0.001 | 0.006 | **Yes** |
| PCB 167 | 0.436 | 0.626 | No |
| PCB 156 | 0.557 | 0.626 | No |
| PCB 169 | 0.717 | 0.782 | No |
| PCB 189 | 0.114 | 0.157 | No |
| PBDE 17 | 0.530 | 0.626 | No |
| PBDE 47 | 0.611 | 0.726 | No |
| PBDE 66 | 0.772 | 0.782 | No |
| PBDE 100 | 0.001 | 0.006 | **Yes** |
| PBDE 153 | 0.584 | 0.726 | No |

*p-values <0.001 were conservatively treated as 0.001 for FDR computation.*

**Table S4** Multivariable-adjusted p-values and Benjamini–Hochberg FDR-corrected p-values for stratified analyses of CYP1A1, GSTP1, GSTM1, GSTT1, and OGG1 polymorphisms according to age, gender, and smoking status.

| **Gene** | **Stratified factor** | **Adjusted p-value** | **FDR-adjusted p-value (BH)** | **Significant after FDR (q<0.05)** |
| --- | --- | --- | --- | --- |
| CYP1A1 | Age | <0.001 | 0.015 | **Yes** |
| OGG1 | Age | <0.001 | 0.008 | **Yes** |
| GSTP1 | Age | <0.001 | 0.005 | **Yes** |
| GSTM1 | Age | <0.001 | 0.004 | **Yes** |
| GSTT1 | Age | 0.001 | 0.003 | **Yes** |
| GSTP1 | Gender | 0.102 | 0.255 | No |
| GSTM1 | Gender | 0.122 | 0.261 | No |
| OGG1 | Gender | 0.143 | 0.268 | No |
| CYP1A1 | Gender | 0.194 | 0.323 | No |
| GSTT1 | Gender | 0.251 | 0.376 | No |
| CYP1A1 | Smoking | 0.688 | 0.938 | No |
| OGG1 | Smoking | 0.743 | 0.929 | No |
| GSTM1 | Smoking | 0.743 | 0.857 | No |
| GSTT1 | Smoking | 0.747 | 0.800 | No |
| GSTP1 | Smoking | 0.753 | 0.753 | No |

**Table S5** Comparison of Tissue POP Concentrations Between the Present Study and Published Reports. A, Omentum control OCP levels; B, Omentum cancer OCP levels; C, Omentum control PCB levels; D, Omentum cancer PCB levels; E, Gastric control OCP levels; F, Gastric cancer OCP levels; G, Gastric control PCB levels; H, Gastric cancer PCB levels.

Top of Form

**Fig. S1** Median omental adipose tissue levels of individual and total POPs in the control and cancer groups. The numbers in parentheses below the group labels indicate the number of individuals in whom each POP was measurable (first number) and the total number of individuals in the group (second number). The median values and levels of significance are shown above and below each graph, respectively. A, omental tissue OCP levels; B, omental tissue non-dioxin-like PCB levels; C, omental tissue dioxin-like PCB levels; D, omental tissue PBDE levels.

**Fig. S2** Significant cross-correlations between blood, gastric, and omental adipose tissue POP levels in the cancer group are depicted. Correlation coefficients and p-values are presented in each graph. The POPs are arranged from the strongest to the weakest correlation in terms of significance. Panel A represents blood and tumor tissue cross-correlations, Panel B represents blood and omental adipose tissue cross-correlations, and Panel C represents tumor and omental adipose tissue cross-correlations.

**Fig. S3** Significant cross-correlations between gastric tissue POP levels and CYP1A activities in control and cancer groups. Correlation coefficients and p values were shown in each graph. The POPs are aligned from strongest to the weakest correlation in terms of significance
